# Immunosuppressive/anti-inflammatory cytokines directly and indirectly inhibit endothelial dysfunction- a novel mechanism for maintaining vascular function

**DOI:** 10.1186/s13045-014-0080-6

**Published:** 2014-10-31

**Authors:** Ying Shao, Zhongjian Cheng, Xinyuan Li, Valeria Chernaya, Hong Wang, Xiao-feng Yang

**Affiliations:** Department of Pharmacology, Center for Metabolic Disease Research and Cardiovascular Research Center, Temple University School of Medicine, MERB 1059, 3500 North Broad Street, Philadelphia, 19140 PA USA; Department of Microbiology and Immunology, Temple University School of Medicine, Philadelphia, 19140 PA USA

**Keywords:** Anti-inflammatory cytokines, Endothelial dysfunction, Metabolic cardiovascular diseases

## Abstract

Endothelial dysfunction is a pathological status of the vascular system, which can be broadly defined as an imbalance between endothelium-dependent vasoconstriction and vasodilation. Endothelial dysfunction is a key event in the progression of many pathological processes including atherosclerosis, type II diabetes and hypertension. Previous reports have demonstrated that pro-inflammatory/immunoeffector cytokines significantly promote endothelial dysfunction while numerous novel anti-inflammatory/immunosuppressive cytokines have recently been identified such as interleukin (IL)-35. However, the effects of anti-inflammatory cytokines on endothelial dysfunction have received much less attention. In this analytical review, we focus on the recent progress attained in characterizing the direct and indirect effects of anti-inflammatory/immunosuppressive cytokines in the inhibition of endothelial dysfunction. Our analyses are not only limited to the importance of endothelial dysfunction in cardiovascular disease progression, but also expand into the molecular mechanisms and pathways underlying the inhibition of endothelial dysfunction by anti-inflammatory/immunosuppressive cytokines. Our review suggests that anti-inflammatory/immunosuppressive cytokines serve as novel therapeutic targets for inhibiting endothelial dysfunction, vascular inflammation and cardio- and cerebro-vascular diseases.

**Electronic supplementary material:**

The online version of this article (doi:10.1186/s13045-014-0080-6) contains supplementary material, which is available to authorized users.

## Introduction

The endothelium has long been viewed not only as a monolayer of endothelial cells (EC) lining the lumen of all blood vessels to function as a protective biocompatible barrier between tissues and circulating blood, but also as a highly specialized, heterogeneous [1], dynamic, disseminated organ [2] with paracrine and autocrine functions which respond to alterations of hemodynamic forces and chemical stimuli. In its entirety, the endothelium is composed of 1 to 6 × 10^13^ EC covering a surface area of more than 1,000 square meters [3],[4]. Endothelial dysfunction is a systemic pathological condition, which can be defined as an imbalance between endothelium-dependent vasoconstriction and vasodilation. Endothelial dysfunction initiates a number of events that trigger EC activation, which predisposes the vessel wall to be stimulated by cardiovascular risk factors [5]. Specifically, endothelial dysfunction is associated with reduced nitric oxide (NO) production, increased reactive oxygen species (ROS) production, upregulation of cytokines and chemokines, decreased anticoagulant properties and enhanced platelet aggregation and leukocyte adherence. One related but more specific term known as endothelial activation is characterized by the upregulation of EC adhesion molecules such as intercellular adhesion molecule-1 (ICAM-1), vascular cell adhesion molecule-1 (VCAM-1) and increased secretion of cytokines and chemokines, which facilitates trans-EC migrations of monocytes, macrophages, dendritic cells, leukocytes, B cells, natural killer cells, and T cells [6]. Perturbations in EC functions play important roles in the development of several major diseases including cardiovascular diseases, metabolic syndrome, systemic inflammatory diseases, and sepsis [7].

**Figure 1:**
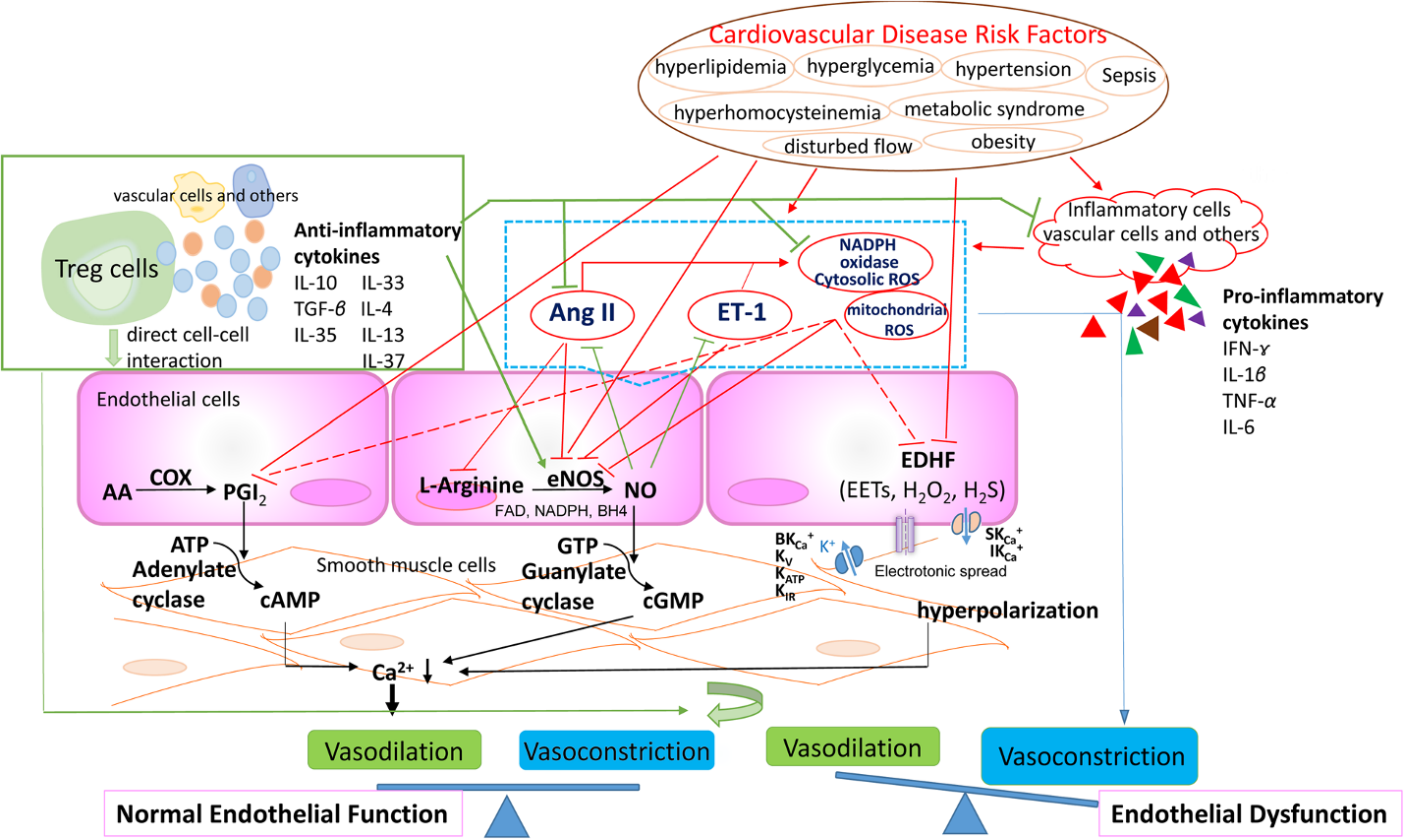
**A new working model: Regulatory T cells and immunosuppressive/anti-inflammatory cytokines inhibit endothelial dysfunction.** In physiological status, the interaction between endothelium-dependent vasoconstrictors (including Ang II, ET-1, ROS) and vasodilators (NO, EDHF and PGI2) maintain the endothelial function and equilibrium of vascular tone. The impairment of the balance between the vasoconstrictors and vasodilators is generally defined as the endothelial dysfunction. Under cardiovascular diseases risk factors stimuli, when vasodilation pathways being impaired or vasoconstriction being activated, endothelial dysfunction occurs. Regulatory T cells (Tregs) act on endothelial cells via cell-cell-interaction and/or immunosuppressive/antiinflammatory cytokines to inhibit endothelial dysfunction and restore normal vascular tone.

Cytokines, including lymphocyte-generated lymphokines, monocyte-produced monokines, chemokines [8], interferons, interleukins, adipocyte-secreted adipokines [9] and muscle-generated myokines [10] act by binding to their specific receptors in concert with specific cytokine inhibitors and soluble cytokine receptors, to regulate innate and adaptive immune responses. They are produced by many types of cells such as vascular EC, in response to the stimulations caused by metabolite-related danger signal-associated molecular patterns (DAMPs) [11] and pathogen-associated molecular patterns (PAMPs) [12] that include bacterial endotoxins, injury, or inflammatory mediators [13]. Cytokines can be divided into pro-inflammatory and anti-inflammatory (immunosuppressive) cytokines. Anti-inflammatory cytokines may either inhibit pro-inflammatory cytokine synthesis or control pro-inflammatory cytokine-mediated cellular activities [14],[15]. Numerous reviews have been published on the roles of pro-inflammatory cytokines on eliciting endothelial dysfunction [16]-[26] and many pro-inflammatory cytokine antagonists have been developed. These new cytokine anta-gonists include: *a)* etanercept, infliximab, adalimumab and certolizumab pegol as tumor necrosis factor-α (TNF-α) antagonists; *b)* Sant1, Sant5, and Sant7 as inter-leukin-6 (IL-6) receptor superantagonists; *c)* anakinra as IL-1 receptor antagonist; *d)* IL-1 receptor antagonist (IL-1Ra) as IL-1α and IL-1α antagonists; *e)* IL-18BP as IL-18 antagonist, and *f)* soluble Endoglin as transforming growth factor-β (TGF-β) antagonist [27]. Potential the-rapeutic effects of these new inflammatory cytokine antagonists on endothelial dysfunction remain unclear. In addition, previous reports showed that anti-inflammatory cytokines IL-10, members of the TGF-β family, and pro-resolving lipid mediators (such as lipoxins, resolvins, and protectins) may suppress pro-inflammatory signaling [28]. Moreover, recent studies have shown that the spectrum of anti-inflammatory cytokinesis expanding [29], which may hold promise for developing potential novel therapeutics and tools for the diagnosis and management of diseases [27]. However, the roles of anti-inflammatory cytokines in endothelial dysfunction have not been extensively analyzed. Therefore, the protective effects of anti-inflammatory cytokines against endothelial dysfunction are the focus of this review (Figure 1).

### Endothelium-dependent regulation of vascular tone

Recent studies have demonstrated that endothelial dysfunction is a mechanistic link between atherosclerotic risk factors and an early development of vascular diseases, which is an independent predictor of future cardiovascular pathologies in patients with atherogenic risk factors or ischemic heart disease [30]. Although endothelial dysfunction may have a more generalized definition, current practical definition is related to the endothelial dysfunction of vascular tone. A healthy endothelium modulates vascular tone by producing numerous vascular dilators and constrictors. In clinical practices, flow-mediated dilation test is the standard tool used to assess endothelial function [31]. In addition, various reactivity tests, coupled with techniques measuring skin blood flux, are used to non-invasively explore both endothelial and neurovascular microvascular functioning in humans [32].

First described by Mulvany and Halpern, vascular tone is measured by using myography in mouse models [33]. This instrument is used for the examination of isolated arteries, with internal diameters between 100–400 μm that are independent of homeostatic mechanisms such as blood flow or autonomic nervous control [34]. The conditions near the physiological setting obtained in myograph allows in-depth characterization of intrinsic vascular tone reactions to physiological and pharmacological stimuli [35]. According to active tension-length relation, force production and the sensitivity of arteries to different agonists are dependent on the extent of stretch [36]. There are two types of myographs for measuring vascular function and studying vascular tones known as the pressure myograph and wire myograph (also see DMT website for an introduction http://www.dmt.dk/default.asp?Action=Details%26Item=543). Pressure myograph may have some advantages over wire myograph such like: *1)* micro resistance arteries with internal diameters of less than 500 μm [37] can be studied whereas wire myograph is limited to large conduit arteries; *2)* the risk to damage endothelium by wire is reduced; *3)* the nature morphology of the arteries is maintained; and *4)* the potential effects of a wide range of pressures and shear stress on artery dimension can be studied [35]. In addition, studying microarteries can be more informative than examining larger conduit arteries to understand the pathophysiological and molecular mechanisms underlying altered vascular tone in certain mouse models of cardiovascular diseases such as hypertension [35]. For example, endothelial dysfunction can be found in 2^nd^ order mesenteric arteries but not in the aortic rings of mice fed with a high fat diet for 8 weeks [38]. In vessel rings pre-contracted with phenylephrine (PE; 1 μM) [39], vascular tone reactivity can be grouped into endothelium-dependent reactivity (to acetylcholine, ACh; 10 nM to 33 μM) and endothelium-independent reactivity (to sodium nitroprusside, SNP; 1 nM to 10 μM), therefore we focus our discussion mainly on endothelium-dependent pathways.

Since high myocardial oxygen extraction occurs at basal conditions, additional metabolic demands may be met by increasing the myocardial blood supply. The coronary artery is capable of increasing the basal flow by at least three folds. This maximum increase in coronary blood flow is defined as “coronary artery reserve”. This remarkable feature is regulated by various vasodilators and vasoconstrictors produced by coronary artery endothelial cells [40]. Not limited to coronary artery endothelium, endothelium generally are capable of producing vasodilators such as NO, prostacyclin (PGI_2_), and endothelium-derived hyperpolarizing factor (EDHF) as well as vasoconstrictors such as endothelin-1 (ET-1), angiotensin II (Ang II), and free radicals [2],[41] that maintain the physiologically balanced vascular tone.

### Endothelium-derived vasodilators

#### Nitric oxide

Among several endothelium-dependent pathways that control vascular tone, the NO pathway is the most prominent in macrovasculature. NO is a key endothelium-derived relaxing factor, produced through the conversion of L-arginine to L-citrulline by the endothelial nitric oxide synthase (eNOS). Once produced in EC, NO diffuses freely into the vascular smooth muscle cells (SMC) where it activates soluble guanylyl cyclase (sGC), which in turn catalyzes the formation of cyclic guanosine monophosphate (cGMP) and finally leads to the relaxation of the vascular smooth muscle [42]. NO is synthesized by a family of NO synthases (NOS) that include neuronal NOS (nNOS), eNOS and inducible NOS (iNOS). These three NOS share 50-60% homology at the amino acid sequence and all have an N-terminal oxygenase domain with heme-, L-arginine- and tetrahydrobiopterin (BH4) binding domains, a central calmodulin (CaM)-binding domain, a C-terminal reductase domain with nicotinamide adenine dinucleotide phosphate (NADPH), flavin adenine dinucleotide (FAD), and a flavin mononucleotide (FMN) binding site [43]. Biochemical and mechanical regulations of eNOS activity play an important role in physiological and pathophysiological responses. eNOS enzymatic activity can be regulated at multiple levels including gene transcription, mRNA stability, substrate, co-factor availability, post-translational modifications, and protein-protein interaction with heat shock protein 90(hsp90) and caveolins[44]. Phosphorylation of eNOS can take place at seven different phosphorylation sites at specific tyrosine (Y), serine (S) and threonine (T). The identified Y sites localize to Y81 and Y567, the S sites localize to S114, S615, S633, and S1177, and the T site localizes to T495 (based on the human eNOS sequence nomenclature) [43]. AKT-1-mediated phosphorylation of S615 and S1177 cooperate to enhance NO generation from eNOS at resting calcium levels, although there is a yin-yang relationship between T495 and S1177 with phosphorylation of one being correlated with dephosphorylation of the other. In addition, phosphorylation of eNOS at S114 decreases eNOS activity [43]. Mechanical shear stress activates eNOS by the phosphorylation of the serine residue 1177 and activation of protein kinase B (PKB, AKT), which itself is phosphorylated by phosphatidylinositol-3-kinase (PI_3_K) [45]. Different from the maintained phosphorylation of serine residue of eNOS in shear stress–stimulated cells, serine 1177 phosphorylation of eNOS in bradykinin-stimulated cells is transient. This eNOS activation requires an elevation in intracellular free calcium concentration, formation of a complex with calmodulin and the removal of the inhibitory caveolin from the activated eNOS complex, although NO production is not affected by the components of PI_3_K-AKT pathway [46].

Besides being the main determinant of the basal vascular tone, NO serves as a natural counterpart to the actions of potent endothelium-derived contracting factors such as Ang II and ET-1. In turn, these factors can also regulate the expression of NOS and NO production [47]-[49]. Reduced NO bioavailability, resulting from either insufficient production or increased degradation of NO, is one of the dominant features of endothelial dysfunction. eNOS requires critical cofactors, such as FAD, NADPH and BH4, to facilitate NO production [50]. In the absence of adequate levels of L-arginine or an essential amount of the cofactors, eNOS may become uncoupled and generate ROS including superoxide anions (O_2_^-^), and hydrogen peroxide (H_2_O_2_) instead of nitrogen species [51]. Therefore, the availabilities of L-arginine and eNOS cofactors FAD, NADPH, and BH4 are very important for NO availability and endothelial function.

#### Endothelium-derived hyperpolarizing factor

EDHF pathway also plays a significant role in vascular tone regulation, especially in small resistant vessels. It triggers potassium ions (K^+^) efflux, which prevents voltage-dependent calcium channel activation, leading to a decrease of the intracellular calcium concentration and relaxation of vascular SMC [52]. The chemical identity of EDHF has not yet been identified. In some cases, it has been found that members of the arachidonic acid (AA) derivatives known as eicosatrienoic acids (EETs) can mediate vasodilation. Agonists such as bradykinin stimulate endothelial G protein-coupled receptors (GPCR) provoking an increase in intracellular calcium in the EC, which activates phospholipase A_2_ (PLA2) mediated AA production. AA metabolism by cytochrome P450 2C (CYP4502C) generates EET, which can stimulate calcium dependent potassium (K_Ca_^+^) channels. Open K_Ca_^+^ channels allows for an efflux and accumulation of K^+^ into the myoendothelial space that triggers the transmission of endothelial cell hyperpolarization to the vascular smooth muscle via gap junctions. In turn, smooth muscle cell hyperpolarization results in relaxation by the closing of voltage-gated channels leading to a fall in Ca^2+^ concentration and subsequent vasodilation [53]. Myoendothelial gap junctions play an important role in electrotonic spread hyperpolarization between EC and SMC. In a number of blood vessels in which myoendothelial coupling is strong, such as in the rat mesenteric artery, the EDHF pathway occurs. While in adult rat femoral artery, in which the smooth muscle and endothelial layers are not coupled electrically, EDHF pathway does not occur even though acetylcholine evokes hyperpolarization in the EC [54]. K_Ca_^+^ channels have been divided into three types, small-conductance (SK_-Ca_^+^), intermediate-conductance (IK_Ca_^+^) and large-conductance (BK_Ca_^+^) channels. A combination of apamin (a specific inhibitor of SK_Ca_^+^) and charybdotoxin (a non-selective inhibitor of BK_Ca_^+^, IK_Ca_^+^ and voltage-dependent K^+^-channels), which has been demonstrated to act selectively on endothelium [55], can completely block EHDF-mediated relaxation [56],[57]. SK_Ca_^+^ and IK_Ca_^+^ channels are abundant in EC and their activation clearly engages in EDHF-induced vasodilation [58]. Meanwhile, as a specific inhibitor of BK_Ca_^+^ , iberiotoxin cannot substitute for charybdotoxin or exclude the role of BK_Ca_^+^ in EDHF-mediated response [59]. BK_Ca_^+^ has been identified in SMC along with three other types of K^+^ channels, voltage-dependent K^+^(Kv) channels, ATP-sensitive K^+^(KATP) channels, and inward rectifier K^+^(Kir) channels [60]. In addition, a study found that IK_Ca_^+^ channels are expressed in proliferative SMC rather than normal SMC, which in turn can define the physiological properties of vascular smooth muscle [61].

Besides K^+^ and EETs, H_2_O_2_ also activates calcium-dependent potassium channels and has been demonstrated to function as a pivotal EDHF [62]. H_2_O_2_ plays an important role in coronary auto-regulation in cooperation with NO and adenosine [63]. In mouse mesenteric arteries, catalase can inhibit ACh-induced endothelium-dependent relaxation and cause hyperpolarization by catalyzing the decomposition of H_2_O_2_ to water and oxygen. This suggests H_2_O_2_ may also perform the role of EDHF in mouse mesenteric arteries [64]. However, catalase does not inhibit non-NO-, non-PGI_2_-mediated vasodilation in all arteries [63]-[65]. Upon ACh stimulation in eNOS knockout mice, the endothelium-produced H_2_O_2_ reduces dramatically, which indicates that eNOS is a major source of H_2_O_2_. In addition, a redox variant of NO, nitroxyl anion (NO^-^), has been proved to be an endogenously derived EDHF that can induce preserved vascular relaxation in the aorta from Ang II-treated hypertensive mice [66],[67]. Recently, H_2_S has also been suggested to be an EDHF. H_2_S causes vascular EC and SMC hyperpolarization and vasodilation by activating the KATP, SK_Ca_^+^ and IK_Ca_^+^ channels through cysteine S-sulfhydration [68].

It has long been accepted that, unlike NO, which induces vasodilation in large conductance arteries, EDHF regulates vascular tone and reactivity in small resistance vessels [69]. However, recent *in vivo* studies found that cytochrome P-450-related EDHF is involved in the regulation of the peripheral conduit artery diameter at rest but not in the control of the basal vascular resistance in the healthy human forearm [70]. After inhibition of NO and prostacyclin, the inhibition of EET synthesis further decreases radial arterial blood flow and diameter. This indicates that EETs plays a potential compensatory role in maintaining basal tone when NO availability is diminished. In addition, it has been demonstrated that in hypercholesterolemia K_Ca_^+^ channel-mediated vasodilation compensates for the reduced NO bioavailability [71]. Furthermore, the activity of EDHF is much higher in individuals with multiple risk factors than that in healthy subjects when compared to NO.

#### Prostacyclin

Prostacyclin, also termed as PGI_2_ is also an endothelium-released effective vasodilator, which functions by binding to GPCRs(G-protein-coupled receptors) to stimulate the production of cyclic adenosine monophosphate (cAMP) that subsequently activates protein kinase A (PKA) and leads to smooth muscle relaxation and vasodilation. PGI_2_ also inhibits vasoconstriction, platelet activation and further coagulation by counteracting the effects of the vasoconstrictor thromboxane A2 (TXA_2_). PGI_2_ and TXA_2_ are produced from AA metabolism mediated by cyclooxygenase (COX). There are two isoforms of COX, which mainly differ in their pattern of expression. COX-1 is expressed in most tissues, whereas COX-2 is usually absent but induced by various physiologic stimuli. COX-1 mostly produces TXA2 while the induction of COX-2 is associated with an increase in PGI_2_ production [72]. P38 and P44/42 mitogen-activated protein kinase (MAPK) pathways mediate the induction of COX-2. In recent years, the wildly accepted COX-2-dependent PGI_2_ production pathway has been challenged by the direct measurement of circulating PGI_2_ levels rather than by the use of urinary markers, which unveiled that it is COX-1, not COX-2, that is responsible for PGI_2_ production in healthy individuals [73]. The opposite conclusions engender controversy of PGI_2_ testing [74], although specific COX-2 inhibitors increase the risk of cardiovascular events, which supports that vascular COX-2 is an important protein for maintaining vascular hemostasis [72]-[74].

The three endothelium-dependent vasodilation pathways, NO, EDHF, and PGI_2_ do not appear to be mutually exclusive but act synergistically in a complex manner to maintain the vasculature health. In addition, the roles of these three components may vary among the vascular beds in different sizes and tissue locations [69]. Moreover, different pathways vary in time course. Many studies have shown that EDHF is more important during the earlier phase of endothelial dysfunction while NO and PGI_2_ are primarily responsible for later and more sustained parts of the vasodilator in response to acetylcholine or bradykinin in various arteries [75],[76].

### Endothelium-derived vasoconstrictors

#### Reactive oxygen species

As analyzed in our recent review [77], ROS can be derived from the transfer of electrons to molecular oxygen in the mitochondrial respiratory systems [78],[79] or produced by the activity of NADPH oxidases, which is co-localized with eNOS in subcellular compartments within EC [80]. This observation provides a direct link between NADPH oxidase and the endothelial function in humans. Small and transient amounts of O_2_^-^ can be beneficial for the endothelial function, through activation of eNOS via Src (a homolog gene highly similar to the v-src gene of Rous sarcoma virus)/PI3-kinase/Akt pathway [51]. High levels of ROS generated in pathological situations will reduce NO bioavailability by the binding of O_2_^-^ to form peroxynitrite (ONOO^-^). This ONOO^-^ coupling with NO results in an imbalance of vascular tension. In addition, NADPH-derived H_2_O_2_ impairs endothelial function by amplifying itself in vascular disease [81]. Elevated levels of ROS have been associated with endothelial dysfunction developed in diabetes mellitus, hypertension, hypercholesterolemia, obesity, atherosclerosis [82]-[86] and sepsis [87]. In addition, several inflammatory cytokines are induced by oxidant stress [88],[89] while cytokines themselves in turn lead to increased levels of ROS and reactive nitrogen species (RNS) in acute or chronic inflammation [90].

#### Angiotensin II

Ang II is part of the renin-angiotensin system that causes vasoconstriction, which increases blood pressure. Ang II stimulates Gq protein in vascular SMC, which increases intracellular calcium levels by an IP_3_-dependent mechanism that consequently leads to contraction. In addition, Ang II enhances vascular arginase activity that impairs NO production by decreasing L- arginine availability [91],[92]. It has been demonstrated that the p38 MAPK pathway participates in this arginase upregulation [93]. Moreover, Ang II also increases superoxide production, which leads to eNOS uncoupling [48],[94] in EC. Meanwhile, other studies demonstrated that the activation of the COX-1 pathway is involved in Ang II-induced development of endothelial dysfunction in small resistance arteries [95],[96].

#### Endothelin-1

ET-1, a 21-aa peptide released by EC, is a natural counterpart of NO, which is normally kept in balance by many mechanisms. ET-1 and NO take part in a paracrine regulation of each other, and the release of ET-1 is blocked by endothelium-derived NO [97],[98]. In pathological situations, ET-1 upregulates the expression of caveolae-1, which appears to be a key negative regulator protein for eNOS activity which leads to eNOS inhibition [99]. In addition, ET-1 can also increase ROS production attributing to NO degradation [100]. Selective endothelin receptor type A (ET_A_) and dual (ET_A_ + ET_B_) antagonists improve NO bioavailability and endothelial function in pathological situations. Transgenic mice that overexpress ET-1 specifically in EC (eET-1) have an increase of endothelial dysfunction, vascular remodeling, oxidative stress, and inflammation [100],[101]. Recently, a study demonstrated that ET-1-induced vascular hypertrophy and oxidative stress participate in innate immunity system activation [102],[103]. Overexpression of ET-1 in EC causes an increase in monocytes/macrophage infiltration into adventitia, which is similar to the findings in mice infused with Ang II [104]. Colony stimulation factor-1 (CSF1) deficiency, a gene mutation impairing monocyte and macrophage production and maturation, can prevent this vascular damage. This finding provides evidence of the roles of monocyte/macrophage as well as innate immunity in ET-1-induced vascular injury.

### Role of anti-inflammatory cytokines in endothelial dysfunction

The delicate balance between pro- and anti-inflammatory cytokines determines the net effect of an inflammatory response. Perturbations in this equilibrium can drive the host defense immune response either towards chronic inflammation (pro-inflammatory) or towards healing (anti-inflammatory). Exposure of endothelial cells to pro-inflammatory cytokines leads to transient and reversible endothelial dysfunction [105],[106]. A number of anti-inflammatory treatment strategies improve endothelial function by preventing pro-inflammatory cytokine synthesis. Anti-inflammatory cytokines are a series of immune-regulatory molecules that control the pro-inflammatory cytokines response, which consequently reduces inflammation and promotes healing. In addition, an elevation in the level of anti-inflammatory cytokines can also be found in the development of vascular disease [107], which reflects an early compensatory mechanism and serves as an indicator of pro-inflammatory reactions. Major anti-inflammatory cytokines include IL-1Ra, IL-4, IL-10, IL-11, IL-13 and TGF-β. Several newly found cytokines, such as IL-33, IL-35, and IL-37 also participate in regulating the function of EC. The following will discuss two of these anti-inflammatory cytokines including IL-10 and TGF-β in detail.

#### Interleukin-10

IL-10 is an anti-inflammatory cytokine produced by many types of immune cells, such as monocytes, macrophages, type 2 T helper cells (Th2), mast cells, natural killer (NK) cells, and CD4 + CD25 + Foxp3+ regulatory T cells (Tregs). Its primary biological function is to limit and terminate inflammatory responses and regulate the differentiation and proliferation of several immune cells. IL-10 receptor 1 (IL-10R1) and IL-10R2 are two subunits of the IL-10 receptor that are expressed by hematopoietic and nonhematopoietic cells. The receptor expression has also been observed in endothelial cells [108],[109], which provides the structural evidence for IL-10 to not only counteract pro-inflammatory cytokines but also potentially inhibit endothelial dysfunction directly. Many studies have found that IL-10 is a key mediator of vascular protection in atherosclerosis, type II diabetes and hypertension [110]-[112]. IL-10 is shown to protect endothelial function by initiating the degradation of several cytokine mRNAs, inhibiting the production of monocyte/macrophage- and neutrophil-derived cytokines [113] and attenuating induction of superoxide generation within the vascular wall [114],[115]. Clinically, in patients with coronary artery disease, IL-10 serum level acts as an independent predictor of the endothelium-mediated vasodilator response of the forearm circulation [116]. Furthermore, IL-10 prevents the impairment of endothelial dysfunction induced by elevated levels of C-reactive proteins [116].

IL-10 also attenuates inflammatory responses by its antioxidant properties. It plays a protective role in blood vessels by inhibiting NADPH oxidase activity and ROS production [111],[117]. In addition, IL-10 can restore Ang II-induced endothelium-dependent relaxation impairment measured by wire myographs in healthy murine aorta rings [118]. Mechanistically, Ang II leads to endothelial dysfunction by increasing gp91phox (NOX2) expression, which is a subunit of NADPH oxidase, while IL-10 inhibits this response by normalizing NADPH oxidase protein expression. In IL-10 knockout (IL-10^-/-^) mice, carotid arteries and thoracic aortas show a marked augmentation of vascular dysfunction after systemic treatment with Ang II that can be prevented by the treatment with superoxide dismutase-mimetic compound TEMPOL [112]. In Ang II-infused hypertensive mice, IL-10 that is released by transferred CD4^+^CD25^+^ natural Treg cells from wild type mice significantly reduces NAPDH oxidase activity and systolic blood pressure; while the transfer of Tregs isolated from IL-10^-/-^ mice has no effect on the hypertension mice. Collectively, these results suggest that IL-10 generated by the immunosuppressive Treg cells protects against Ang II-induced vascular dysfunction and hypertension development by suppressing oxidative stress [119].

In recent studies on aging, old IL-10^-/-^ mice are shown to have stiffer vessels and more severe endothelium-dependent relaxation impairment than wild type mice, which can be reversed by a scavenger of superoxide [120]. In addition, gp91phox is significantly induced in IL-10^-/-^ than that of wild type mice in aging vessels. Furthermore, it is demonstrated that IL-10 protects against age-related endothelial dysfunction by the inhibition of oxidative stress. Aside from oxidative stress, it was also found that there is an increase of COX-2 activity and consequently activation in thromboxane A_2_ receptor instead of PGI_2_ in older IL-10^-/-^ mice [121].

*In vitro*, IL-10 suppresses the production of pro-inflammatory cytokines such as interferon-γ (IFN-γ*)*, IL-1β, TNF-α, and IL-6 by immune cells including T cells, monocytes, macrophages and dendritic cells to produce [113],[122]-[124]. Meanwhile, IL-10 blocks the activity of NF-κB [125], which is a key pro-inflammatory transcription factor [126]-[128]. *In vivo*, IL-10 dampens the pro-inflammatory effects of IL-1 and TNF by stimulating IL-1Ra and soluble TNF receptors (sTNFR) production [129]. In TNF-α-treated mouse models, impairment of vascular relaxation is accompanied with a reduction of eNOS, which can be counteracted by IL-10 which induces eNOS expression and attenuates superoxide production [130]. In addition, by using mouse aortic rings in a myograph, TNF-α triggers a significant decrease in ACh-induced relaxation, which can be restored by IL-10.

The Janus kinase/signal transducers and activators of transcription (JAK/STAT) signaling pathway plays an essential role in mediating the anti-inflammatory actions of IL-10 [131]. In human EC, IL-10 up-regulates eNOS expression and activity mediated by the activation of STAT3 [132]. IL-10 and IL-10 receptor interaction involves the JAK family tyrosine kinases Jak1 and Tyk3 that induce tyrosine phosphorylation and the activation of latent transcription factors STAT3, STAT1, and STAT5 [133],[134]. IL-10 inhibits pro-inflammatory cytokine production in the macrophages via a JAK/STAT3-dependent pathway [135]. In IL-10^-/-^ mice, STAT3 phosphorylation induction does not occur.

Aside from the JAK/STAT pathway, recent studies suggest that IL-10 also confers endothelial protection through several other signaling pathways. IL-10 inhibits the IL-1-induced inhibitor of kappa B (IκB) expression, decreases IκB phosphorylation and causes an increase in the eNOS activity [136]. In addition, IL-10 is associated with the inhibition of extracellular signal-regulated protein kinases 1 and 2 (ERK1/2) activity and the MAPK kinase (MEK)/ERK pathway [137]-[139]. In TNF-α-infused IL-10^-/-^ mice there is an increase of total and phosphorylated ERK1/2 [140], and the aorta and mesenteric arteries isolated from those mice display increased contractile responses to ET-1 through the ET_A_ receptor, which can be abrogated by the ERK1/2 inhibitor PD-98059. This result demonstrates that IL-10 attenuates ET-1 induced vascular injury through the inhibition of the ERK1/2 pathway.

Emerging evidence also suggests that IL-10 plays a major role in suppressing endothelial dysfunction in lipopolysaccharide (LPS)-induced endotoxemia. In LPS-induced endotoxemia, activated ERK1/2 can induce higher expression of IL-10. IL-10 in turn restores eNOS-mediated relaxation by inhibiting production of ROS in monocytes and neutrophils. Meanwhile, it also decreases the production of pro-inflammatory cytokines such as TNF-α and IL-6, leading to an attenuation of septic shock [141]. Mechanistically, IL-10 inhibits the transcription of several inflammatory genes that are induced by the Toll-like receptor (TLR) signaling, such as COX-2, IL-8, and IL-1 [142],[143]. This response can be completely or partially abrogated by the PI3K or Akt1/2 inhibitor. The PI3K-Akt-glycogen synthase kinase 3 (GSK3) pathway regulates IL-10-induced gene expression and controls the ability of IL-10 to suppress a set of inflammatory genes [144].

In summary, as a cytokine synthesis inhibitor factor (CSIF), IL-10 inhibits a broad spectrum of the functions of activated monocytes/macrophages and T cells, including pro-inflammatory cytokine synthesis, and NO production. It also contributes to an essential part in the balance between pro- and anti-inflammatory cytokines.

### Transforming growth factor-β

TGF-β is a multifunctional growth factor capable of inducing cell proliferation, differentiation, programmed cell death, and stimulating matrix deposition. TGF-β family members are also a set of pleiotropic secreted signaling molecules with unique and potent immunoregulatory properties that not only significantly contribute to establishing and maintaining the vascular wall integrity [145], but also participate in the process of wound healing and tissue fibrosis [146],[147]. Various types of cells, such as all the immune cell lines, secrete TGF-β. There are at least three isoforms including TGF-β1, TGF-β2 and TGF-β3, in which TGF-β1 is predominantly ex-pressed in the immune system and has long been known to mediate remarkable actions in the pathogenesis of many vascular diseases. TGF-β performs anti-inflammatory effects and plays an important role in inhibiting vascular disease. Three types of TGF-β receptors are highly expressed in endothelial cells, in which Type I and II receptors are expressed by all the endothelial cells while Type III receptors are mainly located in microvascular endothelial cells [148]. The expressions of TGF-β receptors in endothelial cells provide the structural evidence for TGF-β to not only counteract pro-inflammatory cytokines but also potentially inhibit endothelial dysfunction directly. Moreover, a significant increase in eNOS mRNA levels can be found in TGF-β treated EC [149]. In D-glucose stimulated human umbilical vein endothelium cells (HUVEC), TGF-β binds to type II TGF-β receptors and increases L-arginine transport and NO synthesis, which protects against hyperglycemia-induced endothelial dysfunction [150]. In addition, TGF-β1 knockout mice develop multifocal inflammatory disease associated with increased inflammatory cytokine production, which demonstrates that TGF-β is essential in immune suppression under physiological conditions [151].

In EC, TGF-β increases eNOS expression by activating Smad2, a transcription factor, which interacts with the eNOS promoter [149]. Meanwhile, NO inversely controls the transcription of TGF-β via the endothelial NO/cGMP/PKG pathway and interferes with TGF-β/Smad2 signaling *in vitro* and *in vivo*[149],[152]. With NO donor treatment, EC show a decreased response to TGF-β and suppressed TGF-β target gene expression. Reversely, in eNOS deficient mice, the impairment of NO signaling leads to the upregulation and activation of TGF-β, which accelerates vascular injuries. These results show that the TGF-β dependent effects are complex and can be modulated by NO bioavailability. In response to shear stress, TGF-β3 plays a protective role in maintaining EC homeostasis accompanied by eNOS phosphorylation that leads to the release of NO [153]. In a high salt intake mouse model, TGF-β induced by an excess salt diet restores endothelial NO production via AKT activation and NOS3 phosphorylation as well as alleviates arterial compliance impairment, which forms an inhibitory feedback loop during the vascular function alteration [154].

The inhibitory effects of TGF-β are also in association with Tregs, which perform important immunosuppressive effects [155]. TGF-β converts CD4^+^CD25^+^ effector T cells into CD4^+^CD25^+^ Treg cells by inducing Foxp3 expression. TGF-β signaling is not only required for the survival of peripheral Tregs, but also for the maintenance of Treg suppressive function. Further studies found that TGF-β1 secreted by effector T cells is dispensable for the development and maintenance of Treg cells, while local TGF-β1 production from infiltrating Treg cells appears to be required for Treg immunosuppressive functions [156]. It is well known that Treg cells play an active role in the prevention of cardiovascular diseases [157]. Treg adoptive transfer prevents Ang II–induced hypertension and alleviates aldosterone-induced impairment of the vasodilatory response of resistance mesenteric arteries to Ach [104]. The NOS inhibitor significantly decreases the protective effects of Treg cells, indicating that Treg cells confer protection against the Ang II induced vasodilatory responses through a NO-dependent pathway. In addition, Ang II–induced NADPH oxidase activity can be prevented by Treg adoptive transfer. Thus, the suppressive function in inhibiting both macrophages and T lymphocytes from Tregs is associated with Tregs' contribution to the inhibition of Ang II–induced oxidative stress.

All together, there is a considerable interest in TGF-β for its potential role to be used as a therapeutic target, which prevents endothelial dysfunction through either inducing NO production or counteracting oxidative stress.

#### Other anti-inflammatory cytokines

IL-37, previously known as IL-1 family member 7, has been identified as a new anti-inflammatory cytokine. It is expressed in several tissues and inflammatory cells. Mice with transgenic expressions of IL-37 are protected from LPS-induced endotoxemia and show markedly reduction of liver damage and improved lung and kidney function [158]. Meanwhile, through the interaction with intracellular smad3, IL-37 significantly decreases the expression of various pro-inflammatory cytokines, such as IL-6, IL-1β, IL-17 and IFN-γ both in plasma and organs when compared with vehicle-treat control mice. It has also been observed that IL-37 suppresses the production of pro-inflammatory cytokines in macrophages and epithelial cells, but its role in regards to EC is yet to be explored.

IL-33, also a member of the IL-1 cytokine family, is another newly identified anti-inflammatory cytokine [159]. IL-33 shows various protective effects in the cardiovascular system when ligated with ST2 (IL-33 receptor), a member of the Toll-interleukin 1 receptor (TIR) superfamily. IL-33 markedly decreases the aortic sinus atherosclerotic lesion size in apolipoprotein E (ApoE)^−/−^ mice fed on a high fat diet, which is accompanied by the induction of IL-4, IL-5, and IL-13 and reduction of IFN-γ in the serum [160]. In type II diabetes, IL-33 shows protective metabolic effects by improving insulin tolerance, as well as reducing fasting glucose and adiposity [161]. So far, the molecular mechanisms underlying IL-33's protective effects are not fully understood. Recently, it was found that IL-33 is widely expressed in normal human tissues such as branched blood vessels, lymphoid tissues, adipocytes, cardiac fibroblasts, and even in human tumors. As a novel nuclear marker, IL-33 has been identified as an endogenous alarm in the immune system when the endothelium gets injured during infection and stress [162].

IL-4 and IL-13 both decrease the sensitivity of vascular EC to complement-mediated killing and apoptosis through the activation of a PI_3_K/Akt Pathway [163]. In addition, IL-4 protects EC from complement injury by upregulating claudin-5 through JAK/STAT6 and FoxO1 activation [164].

As a member of the IL-12 family, IL-35 has been identified as a novel anti-inflammatory/immunosuppressive cytokine generated by Tregs [165] and B cells [166],[167]. We found that unlike IL-10 and TGF- β, IL-35 is not constitutively expressed in tissues and is mainly produced by inflammatory stimuli in EC, SMC and monocytes [168]. IL-35 also triggers CD4^+^CD25^-^ T effector cell transformation into CD4^+^CD25^+^ independent of Foxp3 expression. However, the potential effect of IL-35 on endothelial dysfunction remains unknown.

#### MicroRNA and endothelial dysfunction

MicroRNAs (miRNAs) are a recently discovered class of posttranscriptional modulators of gene expression that have an essential role in vascular diseases [169],[170]. They are a group of highly conserved, small, non-coding RNAs that, after maturation and entry into the RNA interference pathway, inhibit specific gene expression. Specific microRNAs can be regulated by inflammatory stimuli and certain microRNAs can act as mediators of inflammatory stimuli. In a human acute monocytic leukemia cell line, LPS stimulates the expression of *miR-146a*, *miR-132* and *miR-155.* Overexpression of *miR-146a* inhibits not only the expression of interleukin-1 receptor-associated kinase and TNF receptor-associated factor 6's [171], but also the expression of IL-6 and IL-8 [172],[173]. In turn, the induction of *miR-155*, which is mediated by NFκB and the activator protein-1 (Ap-1) pathway, inhibits the expression of IL-8 [174],[175], which ultimately demonstrates a negative feedback loop involving microRNAs in an inflammatory response. Some miRNAs were proven to be highly expressed in EC *in vitro*[176]. TNF induced miR-31, miR-17-3p and miR-126 significantly increase the expression of E-selectin, ICAM-1 and VCAM-1 in EC [177],[178]. Transfections with mimics of these miRNAs decrease neutrophil adhesion to EC [177]. *miR-181b*, which is decreased by TNF, can down-regulate the NF-kB-responsive genes such as VCAM-1 and E-selectin in EC *in vitro* and *in vivo*[179]. In miR-10a knockdown human aortic EC, MCP-1 (monocyte chemotactic protein 1), IL-6, IL-8, VCAM-1 and E-selectin are highly elevated, which contributes to athero-susceptible phenotypes *in vivo*[180], suggesting that miR-10a suppresses atherogenesis.

In the aspect of regulating vascular tone, a recent report found that miR-222/221 controls eNOS protein levels after a member of the RNase III family know as dicer is knockdown [181]. Induced by shear stress, miR-21 can stimulate the phosphorylation of NOS and thereby increase NO production [182]. In contrast, miR-155 causes a reduction of NO by decreasing NOS expression [183]. In addition to mediating NO-derived vasodilation, microRNAs also play a role in regulating vasoconstriction. A recent report [184] found that miR-125a/b-5p can suppress oxidized low density lipoprotein (oxLDL) induced ET-1 expression by directly repressing prepro-ET-1 mRNA expression.

In summary, working together with the classical anti-inflammatory cytokines, anti-inflammatory/anti-atherogenic microRNAs [169],[170], as a new concept we discussed, could be an important therapeutic target for protecting against endothelial dysfunction and controlling cardiovascular diseases.

## Conclusions

Endothelial dysfunction has been known as a well-established response to cardiovascular risk factors and precedes the development of cardiovascular diseases. Anti-inflammatory cytokines protect against the impairment of endothelial function by counteracting the effects of pro-inflammatory cytokines and suppressing oxidative stress. Further studies performed on the inhibitory properties of anti-inflammatory cytokines on endothelial dysfunction may provide novel promising therapeutic strategies for the treatment of cardiovascular diseases.

## Authors' contribution

YS carried out the primary literature search and drafted the manuscript. ZC, XL, and VC provided material input and helped revising the manuscript. HW and XFY supervised the manuscript writing and provided field expertise. All authors read and approved the final manuscript.

## Authors’ original submitted files for images

Below are the links to the authors’ original submitted files for images. Authors’ original file for figure 1

## Abbreviations

AA: Arachidonic acid
Ach: Acetylcholine
Ang II: Angiotensin II
ATP: Adenosine triphosphate
BH4: Tetrahydrobiopterin
BK_Ca_^+^: Large-conductance channels
cAMP: Cyclic adenosine monophosphate
cGMP: Cyclic guanosine monophosphate
COX: Cyclooxygenase
EC: Endothelial cells
EDHF: Endothelium-derived hyperpolarizing factor
EETs: Eicosatrienoic acids
eNOS: Endothelial nitric oxide synthase
ERK1/2: Extracellular signal-regulated protein kinases 1 and 2
ET-1: Endothelin-1
ET_A_: Endothelin receptor type A
FAD: Flavin adenine dinucleotide
GTP: Guanosine triphosphate
GPCRs: G-protein-coupled receptors
H_2_O_2_: Hydrogen peroxide
ICAM-1: Intercellular adhesion molecule-1
IFN-γ: Interferon-γ
I-B: Inhibitor of kappa B
IK_Ca_^+^: Intermediate-conductance channels
IL-1Ra: IL-1 receptor antagonist
IL-10^-/-^: IL-10 knockout
JAK: JANUS kinase
K^+^: Potassium ions
K_ATP_: ATP-sensitive K^+^(K_ATP_) channels
K_Ca_^+^: Calcium dependent potassium channels Kir, inward rectifier K^+^ channels
Kv: Voltage-dependent K^+^ channels
LPS: Lipopolysaccharide
MAPK: Mitogen-activated protein kinase
MiRNAs: MicroRNAs
NADPH: Nicotinamide adenine dinucleotide phosphate
NO: Nitric oxide
O_2_^−^: Superoxide anions
ONOO^−^: Peroxynitrite
PI_3_K: Phosphatidylinositol-3-kinase
PGI_2_: Prostacyclin
ROS: Reactive oxygen species
STAT: Signal transducers and activators of transcription
SK_Ca_^+^: Small-conductance channels
SMC: Smooth muscle cells
TGF-β: Transforming growth factor-β
TNF-α: Tumor necrosis factor-α
Treg: Regulatory T cells
TXA_2_: Thromboxane A2
VCAM-1: Vascular cell adhesion molecule-1
