# Supplementary figures and images for: Immunosuppressive/anti-inflammatory cytokines directly and indirectly inhibit endothelial dysfunction- a novel mechanism for maintaining vascular function
Source: J Hematol Oncol. 2014 Oct 31;7:80. doi: 10.1186/s13045-014-0080-6 (PMC4236671; doi:10.1186/s13045-014-0080-6)

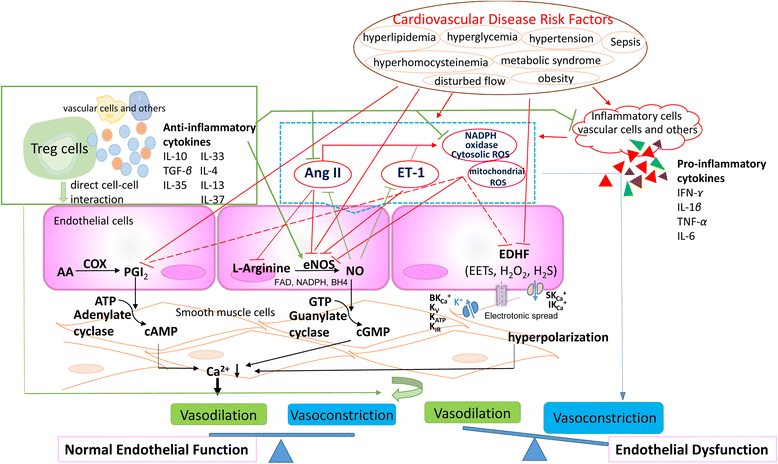

Supplement: Supplementary file 1 — Authors’ original file for figure 1 [file 13045_2014_80_MOESM1_ESM.gif]
